# The Century Magazine

**Published:** 1884-06-20

**Authors:** 


### *THE CENTURY MAGAZINE*

Is justly regarded as among the best, if not the very best, of all the excellent and varied literary publication in the United States. The June No. now before us contains 158 large double-column pages, filled with nu nerous instrnctive, interesting and ably written articles by standard writers, with topics of the time, open letters, bric a brac, etc. It is beautifully illustrated and neatly an handsomely gotten out in all respects. It is published by the Century Co., No. 33 E. Seventeenth Street, New York, at $4.00 per annum, which is certainly very cheap for so large and so admirably magazine.

